# International consensus on initial screening and follow-up of asymptomatic *SDHx* mutation carriers

**DOI:** 10.1038/s41574-021-00492-3

**Published:** 2021-05-21

**Authors:** Laurence Amar, Karel Pacak, Olivier Steichen, Scott A. Akker, Simon J. B. Aylwin, Eric Baudin, Alexandre Buffet, Nelly Burnichon, Roderick J. Clifton-Bligh, Patricia L. M. Dahia, Martin Fassnacht, Ashley B. Grossman, Philippe Herman, Rodney J. Hicks, Andrzej Januszewicz, Camilo Jimenez, Henricus P. M. Kunst, Dylan Lewis, Massimo Mannelli, Mitsuhide Naruse, Mercedes Robledo, David Taïeb, David R. Taylor, Henri J. L. M. Timmers, Giorgio Treglia, Nicola Tufton, William F. Young, Jacques W. M. Lenders, Anne-Paule Gimenez-Roqueplo, Charlotte Lussey-Lepoutre

**Affiliations:** 1 Paris University, Hypertension unit, Hôpital Européen Georges Pompidou, AP-HP, Paris, France; 2 INSERM, PARCC, Equipe Labellisée par la Ligue contre le Cancer, Paris, France; 3 Eunice Kennedy Shriver NICHD, NIH, Bethesda, MD USA; 4 Sorbonne University, Department of Internal Medicine, Hôpital Tenon, AP-HP, Paris, France; 5 St Bartholomew’s Hospital, Barts Health NHS Trust, London, UK; 6 King’s College Hospital NHS Foundation Trust, London, UK; 7 Gustave Roussy Institute and Paris Saclay University, Villejuif, France; 8 Genetics Department, Hôpital Européen Georges Pompidou, AP-HP, Paris, France; 9 Department of Endocrinology, Royal North Shore Hospital, St Leonards, NSW Australia; 10 Sydney Medical School, Faculty of Medicine and Health, University of Sydney, Sydney, NSW Australia; 11 Department of Medicine, Mays Cancer Center, University of Texas Health Science Center at San Antonio, San Antonio, TX USA; 12 Department of Internal Medicine, Division of Endocrinology and Diabetes, University Hospital, University of Würzburg, Würzburg, Germany; 13 Oxford Centre for Diabetes, Endocrinology and Metabolism, Churchill Hospital, Oxford, UK; 14 NET Unit, Royal Free Hospital, London, UK; 15 Centre for Endocrinology, Barts and the London School of Medicine, London, UK; 16 ENT unit, Lariboisière Hospital, AP-HP, University of Paris, Paris, France; 17 Peter MacCallum Cancer Centre, The University of Melbourne, Melbourne, VIC Australia; 18 Department of Hypertension, National Institute of Cardiology, Warsaw, Poland; 19 Department of Endocrine Neoplasia and Hormonal Disorders, The University of Texas MD Anderson Cancer Center, Houston, TX USA; 20 Department of ENT, Radboud University Medical Center, Nijmegen, Netherlands; 21 Maastricht University Medical Center, Maastricht, Netherlands; 22 Department of Experimental and Clinical Biomedical Sciences, University of Florence, Florence, Italy; 23 Endocrine Center, Ijinkai Takeda General Hospital and Clinical Research Institute, NHO Kyoto Medical Center, Kyoto, Japan; 24 Hereditary Endocrine Cancer Group. Spanish National Cancer Research Center (CNIO), Madrid, Spain; 25 Centro de Investigación Biomédica en Red de Enfermedades Raras, Madrid, Spain; 26 Aix-Marseille University, La Timone university hospital, European Center for Research in Medical Imaging, Marseille, France; 27 Department of Internal Medicine, Radboud University Medical Center, Nijmegen, Netherlands; 28 Ente Ospedaliero Cantonale, Bellinzona, Switzerland; 29 Faculty of Biomedical sciences, Università della Svizzera Italiana, Lugano, Switzerland; 30 Faculty of Biology and Medicine, University of Lausanne, Lausanne, Switzerland; 31 Division of Endocrinology, Diabetes, Metabolism, and Nutrition, Mayo Clinic, Rochester, MN USA; 32 University Hospital Carl Gustav Carus, Technische Universität Dresden, Dresden, Germany; 33 Sorbonne University, Nuclear medicine department, Pitié-Salpêtrière Hospital, AP-HP, Paris, France

**Keywords:** Molecular medicine, Adrenal tumours

## Abstract

Approximately 20% of patients diagnosed with a phaeochromocytoma or paraganglioma carry a germline mutation in one of the succinate dehydrogenase (*SDHx*) genes (*SDHA*, *SDHB*, *SDHC* and *SDHD*), which encode the four subunits of the SDH enzyme. When a pathogenic *SDHx* mutation is identified in an affected patient, genetic counselling is proposed for first-degree relatives. Optimal initial evaluation and follow-up of people who are asymptomatic but might carry *SDHx* mutations have not yet been agreed. Thus, we established an international consensus algorithm of clinical, biochemical and imaging screening at diagnosis and during surveillance for both adults and children. An international panel of 29 experts from 12 countries was assembled, and the Delphi method was used to reach a consensus on 41 statements. This Consensus Statement covers a range of topics, including age of first genetic testing, appropriate biochemical and imaging tests for initial tumour screening and follow-up, screening for rare *SDHx*-related tumours and management of elderly people who have an *SDHx* mutation. This Consensus Statement focuses on the management of asymptomatic *SDHx* mutation carriers and provides clinicians with much-needed guidance. The standardization of practice will enable prospective studies in the near future.

## Abstract (web summary)

This Consensus Statement discusses the management of asymptomatic individuals with a germline mutation in one of the succinate dehydrogenase (SDHx) genes (*SDHA*, *SDHB*, *SDHC* and *SDHD*), which encode the four subunits of the SDH enzyme. These individuals are at increased risk of phaeochromocytoma and/or paraganglioma.

## Introduction

Mutations in the *SDHx* genes (*SDHA*, *SDHB*, *SDHC* and *SDHD*), which encode the four subunits of the mitochondrial enzyme succinate dehydrogenase (SDH), are associated with a predisposition for developing hereditary phaeochromocytoma and/or paraganglioma (PPGL)^1^. It is currently recommended that all patients with a newly discovered PPGL should be offered genetic counselling^2^. Germline mutations in *SDHx* genes are responsible for approximately 20% of cases of PPGL and can also be associated with the presence of other *SDHx*-related tumours^1–3^. PPGL detection at an early stage has a positive effect on outcomes, including survival^4^. First-degree family members can also benefit from genetic testing but it remains to be determined how to screen and then follow-up the newly detected asymptomatic mutation carriers as no consensus has as yet been established.

The management of asymptomatic carriers of *SDHx* mutations is a true clinical challenge for several reasons. For instance, patients with *SDHB* mutations are highly predisposed to metastatic PPGL and are at risk of developing multiple tumours, which can be widely distributed from the skull base to the pelvic floor. In addition, PPGLs associated with *SDHx* mutations can be non-functional and, therefore, their detection by biochemical testing is not viable. Furthermore, cumulative radiation exposure from imaging examinations should be limited for genetically predisposed asymptomatic young individuals as they will need lifelong monitoring. Additionally, psychological issues, such as anxiety or depression, can arise during follow-up, which affect a person’s well-being and overall quality of life. A relevant subset of people with *SDHx* mutations will probably never develop a tumour related to the mutation and *SDHx* mutations have been associated with other tumours such as renal cell carcinoma (RCC) and gastrointestinal stromal tumours (GIST)^5–8^. Children who are asymptomatic carriers of *SDHx* mutations should receive special attention as their parents are the decision-makers and undertake a considerable responsibility for the follow-up and outcome of their children^9,10^.

As *SDHx*-related PPGLs are rare tumours, current practice is not guided by robust evidence and consequently differs widely among clinical centres based on local experience and opinion. Thus, using the Delphi method, we developed an international consensus on the clinical, biochemical and imaging screening as well as follow-up of asymptomatic adults and children carrying an *SDHx* mutation.

## Methods

This Consensus Statement was compiled following discussions using the Delphi process between December 2018 and November 2019 with four rounds of questionnaires to confront and converge the thoughts and opinions of the expert panel with the objective of coming to a group consensus^11^.

Experts were identified by their long-standing activity in the field of PPGL management through membership in the European Network for the Study of Adrenal Tumours (ENS@T) and/or the Pheochromocytoma and Paraganglioma Research Support Organization (PRESSOR), a consortium of health science professionals around the world dedicated to research in PPGL.

Of the 39 experts invited to participate, 29 responded to the first-round questionnaire and then completed the second, third and fourth (last) rounds. The Delphi panel included 16 endocrinologists and/or internists, 6 imaging specialists, 2 head and neck surgeons and 5 geneticists. The experts are from 20 centres, representing 12 countries across four continents (22 from Europe, 4 from the USA, 2 from Australia and 1 from Asia).

Online software was used to house the questionnaires and responses. The first questionnaire was designed by the two core group members (L.A. and C.L.L.) and approved by an executive committee (J.W.M.L., K.P. and A.P.G.R.). Experts were invited for the first-round questionnaire by e-mail and three reminders were sent within 3 months. Respondents were included in the consensus process and participated in the subsequent three rounds. The first-round questionnaire asked multiple-choice questions on the screening and follow-up of asymptomatic mutation carriers for each of the four genes (*SDHA*, *SDHB*, *SDHD* and *SDHC*), recognizing that mutations in each of these genes have differing penetrance, dominant sites of disease and malignant potential^12^. The questions covered the age at which the first screening should take place and the biochemical and imaging tests to be used for initial tumour screening as well as for follow-up. Two moderators (L.A. and C.L.L.) independently analysed the answers of experts and translated them into a series of statements with a methodologist (O.S.). These statements had to be rated and commented on by each expert independently using a 5-point Likert scale (1, strongly agree; 2, agree; 3, neutral; 4, disagree; 5, strongly disagree) for the second round of the Delphi process. The results of the second round as well as the ratings and comments were sent to the experts who had to once again rate those statements for which no consensus had been previously reached (see next paragraph for the definition of consensus). When needed, statements were reformulated following the comments of the experts. This process was repeated for the third and fourth rounds. The responses and comments remained anonymous, except to the moderators.

Consensus was defined as ≥23 of 29 experts (≥79%) for agreement (Likert scale 1 and 2) or disagreement (Likert scale 4 and 5) for the first and second rounds. For the third and fourth rounds, consensus was defined as ≥20 of 29 experts (≥69%) for agreement (Likert scale 1 and 2) or disagreement (Likert scale 4 and 5) and 80% of agreement (Likert scale 1 and 2) or disagreement (Likert scale 4 and 5) after the exclusion of neutral answers (Likert scale 3). When no consensus was reached after two consecutive rounds due to opposite ratings or a majority of neutral ratings, the statement was removed from the consensus, with the experts’ approval.

Consensus statements were graded as A for statements with ≥23 of 29 experts with agreement or disagreement or as B for statements with ≥20 of 29 experts with agreement or disagreement and ≥80% after exclusion of neutral answers.

## The question rounds

The questionnaire in the first round included 47 open questions (Supplementary Table S1) on initial and follow-up tumour screening in asymptomatic mutation carriers for each of the four genes (*SDHA*, *SDHB*, *SDHD* and *SDHC*), including the minimum age for offering predictive genetic testing and thus initial screening as well as the biochemical and imaging tests to be used for initial and follow-up tumour screenings. The second round contained 69 statements (Supplementary Table S2). Several questions were reformulated and new questions were included in accordance with the experts’ comments during the first round. The third round comprised 45 statements (Supplementary Table S3) and the fourth round included 11 statements (Supplementary Table S4). Overall, 14 expert recommendations were made, as detailed here.

## Initial screening

### Genetic penetrance

The penetrance of *SDHx*-related PPGL is incomplete and varies in asymptomatic *SDHx* mutation carriers (between 8% and 37% for *SDHB* and 38% and 64% for *SDHD*) across several studies^3,13–23^. People with *SDHD* mutations have the highest penetrance, with multiple tumours most frequently located in the head and neck region (parasympathetic), whereas *SDHB* mutations predispose carriers primarily to retroperitoneal PPGL (sympathetic). *SDHB* mutation carriers are at a higher risk of developing metastases than carriers of mutations in any of the other *SDHx* genes^3,13,24,25^. However, the risk of developing a head and neck paraganglioma for a person with an *SDHB* mutation or a phaeochromocytoma in someone with an *SDHD* mutation is still statistically significant over a lifetime^15^. Data regarding *SDHC* mutation carriers are scarce but these carriers seem to present predominantly with non-metastatic head and neck paragangliomas with a lower rate of multiplicity and a lower penetrance than people who have an *SDHD* mutation^3,20,26^. Very little data have been published on the penetrance of *SDHA* mutations yet; one study reported a penetrance of 13%^27^. Moreover, even if some variants can be associated with specific clinical characteristics or penetrance^3,28^, the experts considered that the data are not yet strong enough to personalize screening and follow-up regarding the type of variant. The expert panel analysed each gene separately but this resulted in similar recommendations for initial screening and follow-up for all the *SDHx* genes, except regarding the age of first tumour screening during childhood.

Regarding the particular transmission model of inheritance for the *SDHD* gene, the recommendations were identical to other *SDHx* genes when an *SDHD* mutation is transmitted through the father (*SDHD*-*pi*). However, when the *SDHD* mutation is transmitted through the mother (*SDHD*-*mi*), the development of a PPGL seems to be a rare event (≤5%)^29^.

- Recommendation 1: Tumour screening should be performed after the identification of an *SDHA*, *SDHB*, *SDHC* or *SDHD*-*pi* mutation in an asymptomatic carrier (Grade A).

### Timing of initial screening in childhood

Even if the risk of developing a tumour during childhood (<18 years old) is low, a small number of *SDHB*-related paragangliomas have been reported at age 6 years^13,20,30–35^ and children with an *SDHB* mutation have a higher risk of developing a metastatic paraganglioma than children with mutations in the other *SDHx* genes^32,36^. As surgery remains the only curative treatment, the experts surmised that earlier tumour detection and removal is likely to be associated with improved long-term prognosis. For *SDHD*-*pi* and *SDHC* mutation carriers, some cases have been described during childhood but, in most cases, children were older than 10 years^3,33,35^. For *SDHA* mutation carriers, paragangliomas have been diagnosed in 17-year-old adolescents according to the literature^37^. The experts reported some cases in 13 year olds. Based on this evidence, the experts proposed first screening at an earlier age (6 years old) for asymptomatic *SDHB* mutation carriers than for carriers of mutations in the other *SDHx* genes (10 years old).

- Recommendation 2: During childhood, genetic screening should only be performed if tumour screening would be considered if a mutation was discovered (Grade B).
- Recommendation 3: During childhood, tumour screening should only be performed following the discovery of a mutation (Grade A).
- Recommendation 4: First tumour screening should be performed between 6 and 10 years of age for asymptomatic *SDHB* mutation carriers and between 10 and 15 years of age in asymptomatic *SDHA*, *SDHC* and *SDHD-pi* mutation carriers (Grade A).

### Screening methods

Initial screening should include an assessment of symptoms (Box 1), a clinical examination and blood pressure evaluation. As recommended by current blood pressure guidelines, blood pressure evaluation should rely on ambulatory blood pressure monitoring with a diagnostic blood pressure threshold for the diagnosis of hypertension of either 130/80 mmHg for patients younger than 65 years or 135/85 mmHg for patients older than 65 years, following international guidelines^38,39^.

Biochemical measurements of urinary or plasma levels of metanephrine and normetanephrine should be carried out to detect catecholamine production. Their superiority over catecholamines and vanillylmandelic acid measurements has long been established^40,41^ and they are currently recommended for the diagnosis of PPGL^2^. Plasma or urinary free metanephrines analyses seem to be the best diagnostic test, with an even higher sensitivity and specificity than analyses of deconjugated urinary metanephrines^42^. Although urinary measurement of metanephrines is less convenient, it might confer an advantage in childhood because this method avoids venepuncture. In 2019, age-specific paediatric reference intervals for plasma free metanephrine and normetanephrine were published^43^.

- Recommendation 5: Tumour screening in asymptomatic *SDHA*, *SDHB*, *SDHC* and *SDHD-pi* mutation carriers should include clinical examination: blood pressure measurement (ideally, out-of-office blood pressure measurement during adulthood) and a symptoms and signs questionnaire (Box 1) (Grade A).
- Recommendation 6: Biochemical testing for tumour screening in asymptomatic *SDHA*, *SDHB*, *SDHC* and *SDHD-pi* mutation carriers should include measurements of either plasma or urinary metanephrine and normetanephrine (Grade A).
  - During childhood, the choice between plasma or urinary tests should be left to the clinician and local laboratory availability and expertise (Grade A).
  - During adulthood, measurements of plasma free metanephrine and normetanephrine should be preferred over urinary measurements (Grade A).
- Recommendation 7: Biochemical testing should not include either vanillylmandelic acid or catecholamines in addition to metanephrine and normetanephrine (Grade A).

An important proportion of tumours, especially *SDHx*-related and/or head and neck paragangliomas arising from the parasympathetic system, do not secrete or even produce catecholamines^44,45^. Thus, the surveillance of patients with non-functional paragangliomas requires the use of imaging; there was a strong consensus on the use of MRI for the head and neck region. Indeed, specific imaging protocols have previously been published in the literature^15,46^. For imaging of thoracic, abdominal and pelvic regions, either CT scanning or MRI can be useful. However, to limit cumulative ionizing radiation exposure in asymptomatic children, experts agreed to recommend MRI. Ultrasound is an option for first-line imaging only in children who might not tolerate MRI^47^. For adults, the first-line for imaging of abdominal and pelvic regions is MRI. To increase the detection rate and explore the thoracic region, it was decided to perform functional imaging using PET–CT at the initial screening for adult mutation carriers. PET–CT imaging has some technical advantages over SPECT–CT (single-photon emission CT) as it has shorter uptake times, is a shorter imaging procedure, has less drug interference and a higher resolution^48^. Nevertheless, consensus was only obtained with grade B agreement (Tables 1,2). As PET–CT involves radiation exposure, it was considered that performing one functional imaging scan is of interest because it provides a 3D whole-body examination with a limited radiation dose exposure (7–8 mSv for ^18^FDG-PET–CT, which is of the same order of magnitude as a cervical, thoracic and abdominopelvic CT scan). Systematic use of PET–CT was preferred over conventional thoracic CT scanning because the latter has a poor sensitivity of only 46.2% (19.2–74.9%)^15^. PET–CT was not recommended for first screening during childhood.

**Table 1:** Grading of recommendations for the percentage of agreement or disagreement (R1–R7)

Recommendation	Likert scale (%)					Grade
	1, strongly agree	2, agree	3, neutral	4, disagree	5, strongly disagree	
R1: Tumour screening should be performed after identification of an *SDHA*, *SDHB*, *SDHC* and *SDHD*-*pi* mutation in an asymptomatic carrier	65.5	27.6	3.4	0	3.4	A
R2: During childhood, genetic screening should only be performed if tumour screening would be considered if a mutation was discovered	34.5	41.4	13.8	6.9	3.4	B
R3: During childhood, tumour screening should only be performed following the discovery of a mutation	41.4	44.8	3.4	6.9	3.4	A
R4 for SDHB: First tumour screening should be performed between 6 and 10 years of age for asymptomatic *SDHB* mutation carriers	51.7	37.9	6.9	0	3.4	A
R4 for other genes: First tumour screening should be performed between 10 and 15 years of age in asymptomatic *SDHA*, *SDHC* and *SDHD-pi* mutation carriers	NA	NA	NA	NA	NA	NA
R4 for *SDHA*	13.8	69.0	10.3	6.9	0	A
R4 for *SDHC*	10.3	72.4	6.9	6.9	3.4	A
R4 for *SDHD*	13.8	65.5	10.3	6.9	3.4	A
R5: Tumour screening in asymptomatic *SDHA*, *SDHB*, *SDHC* and *SDHD-pi* mutation carriers should include clinical examination: blood pressure measurement and a symptoms and signs questionnaire	NA	NA	NA	NA	NA	NA
R5 during childhood	51.7	34.5	6.9	6.9	0	A
R5 during adulthood	69.0	27.6	0	3.4	0	A
R6: Biochemical testing for tumour screening in asymptomatic *SDHA*, *SDHB*, *SDHC* and *SDHD-pi* mutation carriers should include measurements of either plasma or urinary metanephrine and normetanephrine	NA	NA	NA	NA	NA	NA
R6a: During childhood, the choice between plasma or urinary tests should be left to the clinician and local laboratory availability and expertise	41.4	44.8	13.8	0	0	A
R6b: During adulthood, measurements of plasma-free metanephrine and normetanephrine should be preferred over urinary measurements	72.4	17.2	10.3	0	0	A
R7: Biochemical testing should not include either vanillylmandelic acid or catecholamines in addition to metanephrine and normetanephrine	48.3	31.0	10.3	3.4	6.9	A

Summary of the recommendations and grading of the experts using the Likert scale. NA, not applicable.

**Table 2:** Grading of recommendations for the percentage of agreement or disagreement (R8–R14)

Recommendation	Likert scale (%)					Grade
	1, strongly agree	2, agree	3, neutral	4, disagree	5, strongly disagree	
R8: Tumour screening in asymptomatic *SDHA*, *SDHB*, *SDHC* and *SDHD-pi* mutation carriers should include imaging	NA	NA	NA	NA	NA	NA
R8 during childhood	51.7	41.4	6.9	0	0	A
R8 during adulthood	27.6	69.0	0	3.4	0	A
R8a for non-thoracic MRI	34.5	44.8	10.3	10.3	0	A
R8a for thoracic MRI	37.9	37.9	13.8	10.3	0	B
R8b for MRI	31.0	48.3	6.9	10.3	3.4	A
R8b for PET–CT	27.6	44.8	10.3	10.3	6.9	B
R8c	24.1	58.6	10.3	6.9	0	A
R8d during childhood	34.5	37.9	13.8	13.8	0	B
R8d during adulthood	55.2	27.6	6.9	6.9	3.4	A
R8e	48.3	41.4	3.4	6.9	0	A
R8f	62.1	34.5	3.4	0	0	A
R9: *SDHA*, *SDHB*, *SDHC* and *SDHD-pi* asymptomatic mutation carriers should be followed-up on a regular basis after a negative initial work-up	72.4	24.1	3.4	0	0	A
R10: During childhood and adulthood, follow-up of *SDHA, SDHB, SDHC* and *SDHD-pi* asymptomatic mutation carriers should include clinical examination (blood pressure measurement, ideally out-of-office, and a symptom questionnaire), the same biochemical investigations as for the initial screening (for example, metanephrine and normetanephrine) and imaging by MRI (head and neck and thoracic, abdominal, and pelvic)	NA	NA	NA	NA	NA	NA
R10 during childhood	55.2	37.9	3.4	3.4	0	A
R10 during adulthood	69.0	24.1	3.4	3.4	0	A
R10a during childhood	34.5	55.2	6.9	3.4	0	A
R10a during adulthood	41.4	48.3	6.9	3.4	0	A
R10b during childhood	41.4	44.8	6.9	6.9	0	A
R10b during adulthood	31.0	58.6	6.9	3.4	0	A
R10c during childhood	27.6	58.6	10.3	3.4	0	A
R10c during adulthood	34.5	48.3	0	17.2	0	A
R10d during childhood	41.4	37.9	6.9	13.8	0	A
R10d during adulthood	48.3	37.9	3.4	6.9	3.4	A
R10e during childhood	51.7	37.9	3.4	6.9	0	A
R11: If an *SDHA*, *SDHB, SDHC* or *SDHD-pi* mutation carrier never developed any tumour related to SDH deficiency and has been asymptomatic all their life, screening tests should be delayed to every 5 years after 70 years of age and follow-up should be stopped at 80 years of age	NA	NA	NA	NA	NA	NA
R11 for a delayed follow-up	13.8	58.6	10.3	17.2	0	B
R11 for end of follow-up	34.5	44.8	6.9	13.8	0	A
R12: Screening should not differ between male and female individuals; however, complete screening should be performed before planning a pregnancy	34.5	44.8	13.8	3.4	3.4	A
R13: Initial screening and follow-up should not differ for asymptomatic mutation carriers whose family members developed metastatic *SDHx*-related PPGL or those with non-metastatic PPGL	31.0	55.2	3.4	6.9	3.4	A
R14: No additional imaging should be performed for RCC, GIST and pituitary adenoma; nevertheless, RCC and GIST should be searched for on imaging performed for PPGL screening	27.6	62.1	6.9	3.4	0	A

Summary of the recommendations and grading of the experts using the Likert scale. GIST, gastrointestinal stromal tumour; NA, not applicable; PPGL, phaeochromocytoma and/or paraganglioma; RCC, renal cell carcinoma.

- Recommendation 8: Tumour screening in asymptomatic *SDHA*, *SDHB*, *SDHC* and *SDHD-pi* mutation carriers should include imaging (Grade A).
  - During childhood, MRI of head and neck and thoracic, abdominal and pelvic regions should be used as first-line imaging for initial tumour screening (Grade A for head and neck and for abdominal and pelvic MRI, Grade B for thoracic MRI).
  - During adulthood, a combination of MRI (head and neck, abdominal and pelvic; Grade A) and PET–CT (Grade B) should be used as first-line imaging for initial tumour screening.
  - The expert panel recommends performing dedicated thoracic cross-sectional imaging only in instances of a PET–CT abnormality (Grade A).
  - Ultrasound should not be used as first-line imaging for initial tumour screening in asymptomatic *SDHA*, *SDHB*, *SDHC* and *SDHD-pi* mutation carriers (Grade B during childhood, Grade A during adulthood). However, some experts underlined the convenience of ultrasound for some young children who will not tolerate MRI.
  - During childhood, functional imaging should not be used for tumour screening as first-line imaging in asymptomatic *SDHA*, *SDHB*, *SDHC* and *SDHD-pi* mutation carriers (Grade A).
  - During adulthood, functional imaging should rely on PET–CT. ^123^I-MIBG and ^111^In-pentetreotide scintigraphy should not be used as first-line imaging studies for initial tumour screening in asymptomatic *SDHA*, *SDHB*, *SDHC* or *SDHD-pi* mutation carriers (Grade A).

Box 1 Symptom and sign questionnaire

- Do you experience headaches?
  - If yes, on average, how often? They occur … times each day/week/month.
- Do you experience severe sweating for unknown reason?
  - If yes, on average, how often? It occurs … times each day/week/month.
- Do you complain of rapid or forceful heartbeat?
  - If yes, on average, how often? It occurs … times each day/week/month.
- Did you measure your blood pressure during symptoms?
  - If yes, was it elevated?
- Do you look pale when you have acute symptoms?
- Did you notice hearing loss or tinnitus?
- Do you notice any voice changes?
- Did you notice any difficulty in swallowing?
- Did you notice any difficulty in lifting your shoulder?
- Did you lose weight unexpectedly?
  - If yes, how many kg or pounds? Over which period?
- Did you notice a neck mass?
  - If yes, since when?

## Follow-up after a first negative initial screening

*SDHx* mutation carriers have a lifelong risk of developing PPGL^49^. Therefore, one initial screening is not sufficient and these individuals should be followed up. Although people who do not have PPGL at initial screening have a reduced risk of developing new tumours during follow-up, they are still at risk of developing these tumours during their lifetime^49^. This risk has led experts to consider that follow-up is mandatory in asymptomatic carriers of an *SDHx* mutation.

- Recommendation 9: *SDHA*, *SDHB*, *SDHC* and *SDHD-pi* asymptomatic mutation carriers should be followed up on a regular basis after a negative initial work-up (Grade A).

### Screening methods

**Fig. 1:**
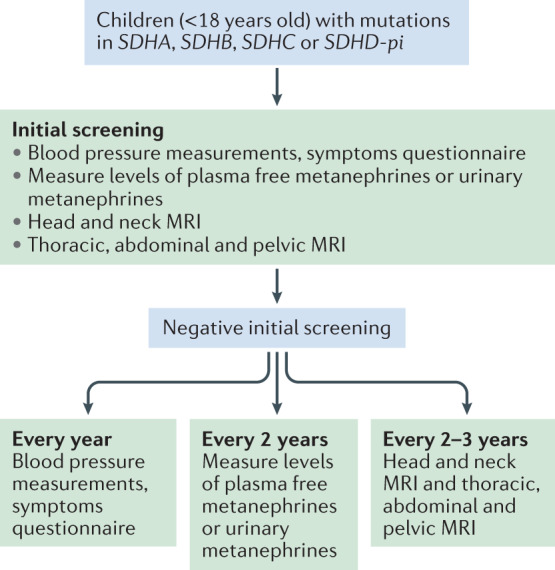
Screening and follow-up proposed during childhood. An initial tumour screening should be performed after the discovery of an *SDHA*, *SDHB*, *SDHC* or *SDHD-pi* mutation relying on blood pressure measurements, a symptoms or signs questionnaire, assessment of metanephrines in plasma or urine, and imaging work-up by MRI of head and neck, thorax, abdomen and pelvis. Even after an initial negative work-up, all asymptomatic mutation carriers should be clinically followed up every year, by biochemical assessments every 2 years and by MRI every 2–3 years.

**Fig. 2:**
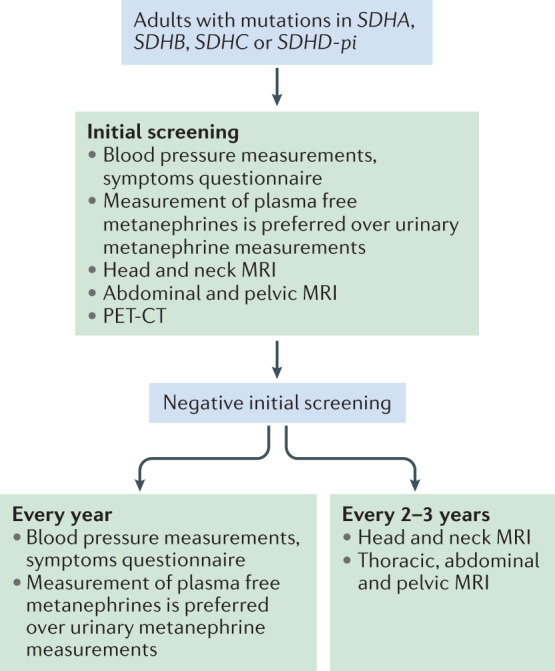
Screening and follow-up proposed during adulthood. An initial tumour screening should be performed after the discovery of an *SDHA*, *SDHB*, *SDHC* and *SDHD-pi* mutation relying on blood pressure measurements, a symptoms or signs questionnaire, assessment of metanephrines in plasma or urine, and imaging work-up by MRI of head and neck, abdomen, pelvis, and a whole-body PET–CT. Even after an initial negative work-up, all asymptomatic mutation carriers should be followed clinically and by biochemistry assessments every year and by MRI every 2–3 years. Thoracic MRI is not mandatory at the first initial work-up if PET–CT does not show any abnormality but is recommended for subsequent follow-up.

The expert committee recommends that all *SDHx* mutation carriers should have an annual outpatient follow-up examination during both childhood and adulthood. Biochemical measurements of plasma or urinary metanephrine and normetanephrine should be conducted every 2 years in children and yearly in adults (Figs 1,2). PET–CT was not recommended for follow-up during childhood.

- Recommendation 10: During childhood and adulthood, follow-up of asymptomatic carriers of *SDHA*, *SDHB*, *SDHC* and *SDHD-pi* mutations should include clinical examination (blood pressure measurement, ideally out-of-office, and a symptom questionnaire), the same biochemical investigations as for the initial screening (for example, metanephrine and normetanephrine) and imaging by MRI (head and neck and thoracic, abdominal and pelvic) (Grade A).
  - Clinical examination should be performed every year (Grade A).
  - Biochemical testing should be performed at least every 2 years during childhood and every year during adulthood (Grade A).
  - MRI should be performed every 2–3 years (Grade A).
  - Ultrasound should not be used as first-line imaging for follow-up in asymptomatic carriers of *SDHA*, *SDHB*, *SDHC* and *SDHD-pi* mutations (Grade A).
  - Functional imaging should not be used for follow-up in asymptomatic *SDHA*, *SDHB*, *SDHC* and *SDHD-pi* mutation carriers during childhood (Grade A). Nevertheless, no consensus emerged for or against alternating PET–CT and MRI imaging during adulthood.

The experts insisted on the fact that the risk of metastatic progression is more important in *SDHB* asymptomatic mutation carriers than for the other genes as many retrospective studies showed a higher risk of metastatic disease and shorter survival in patients with an *SDHB* mutation than in patients with other *SDHx* mutations^3,20,24^.

### End of follow-up

After a certain age, a carrier of an *SDHx* mutation who has not developed a PPGL by initial screening or during follow-up has a considerably reduced risk of developing new tumours^49^.

- Recommendation 11: If an *SDHA*, *SDHB*, *SDHC* or *SDHD-pi* mutation carrier never developed any tumour related to SDH deficiency and has been asymptomatic all their life, screening tests should be delayed to every 5 years after 70 years of age (Grade B) and follow-up should be stopped at 80 years of age (Grade A).

## Additional expert recommendations

It has been established that a secreting PPGL is a perilous condition during pregnancy, with an elevated risk of pre-eclampsia and gestational diabetes and severe cardiac complications for the mother and prematurity and mortality (including miscarriage, intrauterine fetal loss and death at delivery) for the fetus^50^. Therefore, the experts considered that screening should be performed before planning a pregnancy to avoid this dangerous situation in an asymptomatic woman carrying an *SDHx* mutation.

- Recommendation 12: Screening should not differ between male and female individuals. However, complete screening should be performed before planning a pregnancy (Grade A).

Since the discovery of the *SDHx* genes, family screening has been widely performed. There is currently only weak evidence indicating that genotype predicts the underlying phenotype. In the same family with all individuals carrying the same genetic variant, it is possible to see lifelong asymptomatic carriers, patients with a single head and/or neck paraganglioma, and others with metastatic PPGL^21^. Evidence suggests that metastatic progression in *SDHx*-mutated PPGL is due to immortalization-related mechanisms (TERT activation or *ATRX* mutations) occurring in the primary tumour^51^.

- Recommendation 13: Initial screening and follow-up should not differ for asymptomatic mutation carriers whose family members developed metastatic *SDHx*-related PPGL or those with non-metastatic PPGL (Grade A).

*SDHx* mutations have been associated with other tumours with a causal link to RCC and GIST. It remains less clear if *SDHx* mutations can be associated with other tumours that could be found in affected carriers or their relatives such as pituitary adenomas^52–55^.

The majority of GIST in adults are secondary to somatic mutations in the *KIT* and *PDGFRA* genes^56,57^. However, 15% of GIST in adults and 85% of GIST developing during childhood have *SDHx* mutations^58,59^. The majority of *SDHx* mutations identified in GIST are germline *SDHA* point mutations followed by *SDHB* and *SDHC* point mutations^5^. Recurrent epimutations of *SDHC* have been identified in GIST and Carney triad (a syndrome characterized by paraganglioma, GIST and pulmonary chondroma). Most of the epimutations are somatic events without risk of familial transmission^60^. Regarding RCC, *SDHx* mutations appear to be implicated in 0.05–0.20% of cases, mainly affecting *SDHB*^61,62^. In the most recent WHO classification, *SDHx*-related RCC were identified as a new subtype of RCC^63^. In large cohorts of *SDHx* mutation carriers, the penetrance of RCC is estimated at 2–3% of patients^3,33^ and the risk of GIST development has not yet been evaluated. Moreover, GIST and RCC are readily detected on MRI^64–66^.

- Recommendation 14: No additional imaging should be performed for RCC, GIST and pituitary adenoma. Nevertheless, RCC and GIST should be searched for on imaging performed for PPGL screening (Grade A).

## Statements without consensus

A consensus was not reached for several statements (Supplementary Table S5).

The screening of people with *SDHD-mi* mutations lacks a consensus due to limited data in the literature. Except for a few case reports^67–69^, only one prospective study evaluated the risk of developing a tumour in 20 *SDHD-mi* mutation carriers^29^. Of these carriers, only one phaeochromocytoma was observed in a 35-year-old woman with a double loss of heterozygosity in the paternally derived q arm and the maternally derived p arm of chromosome 11. This case was considered too rare by experts to form the basis of recommendations as it involved three unique genetic events. Experts were not able to estimate the cost–benefit ratio of screening and surveillance of *SDHD-mi* mutation carriers and, thus, no consensus was reached.

The consideration of environmental factors (for example, living at a high altitude) was also waived. People living at altitudes above 2,000 m develop head and neck paraganglioma (especially carotid body paraganglioma) with a higher frequency than those at lower altitudes, possibly in response to chronic hypoxia^70–75^. However, no evidence of this effect was found in the context of patients with *SDHx* mutations^76^ and therefore no consensus was reached.

No agreement was reached regarding the measurement of plasma levels of dopamine, 3-methoxytyramine (the O-methylated metabolite of dopamine) and chromogranin A in addition to metanephrine and normetanephrine for tumour screening in asymptomatic *SDHx* mutation carriers. Dopamine production by *SDHB*-related PPGLs might be associated with the development of metastases; however, the measurement of dopamine is not recommended for first-line screening^2,35,77^. Plasma measurement of 3-methoxytyramine has prognostic value in patients with *SDHB*-associated PPGLs^77^ and it could be useful in the detection of dopamine-producing PPGLs and certain non-functional head and neck paragangliomas^78,79^. Chromogranin A could add value as a complementary biomarker in *SDHB*-related sympathetic paraganglioma (which typically secrete normetanephrine); however, it is non-specific and often low in head and neck paragangliomas^80^. The use of specific hormones and new biomarkers to characterize PPGLs and their behaviour as well as the value of screening in asymptomatic individuals are yet to be established.

The discussion regarding imaging methods focused on the ideal balance between the early detection of tumours (which can improve patient management)^4^ and reducing radiation exposure. Experts agreed on a comprehensive initial screening in adults to differentiate asymptomatic individuals from patients with an unknown disease as approximately 20% of asymptomatic individuals have one or several tumours upon initial screening^15^. However, some experts voiced the view that functional imaging should only be performed if MRI shows an abnormality. Thus, no consensus was achieved on the frequency of PET–CT during follow-up. The indication of PET–CT once in a lifetime when a child reaches adulthood was not discussed.

Due to the wide variability in availability and costs of the different tracers, the expert panel decided against a consensus for optimal PET radiopharmaceuticals (^68^Ga-DOTA-somatostatin analogues (^68^Ga-DOTA-SSAs), ^18^F-FDOPA, ^18^F-FDG). However, in 2019, EANM–SNMMI joint guidelines proposed the use of ^68^Ga-DOTA-SSAs PET as the first-choice functional imaging modality in *SDHx* mutation carriers and the use of ^18^F-FDG and/or ^18^F-FDOPA PET when ^68^Ga-DOTA-SSAs PET is not available^81^.

## Conclusions

It is strongly recommended that asymptomatic carriers of *SDHx* mutations (detected by familial genetic testing) undergo regular biochemical testing and clinical examination. Due to the lack of robust data in the literature, a Delphi process enabled the proposal of an expert consensus statement. This consensus on when to begin screening and end follow-up as well as the appropriate imaging for the management of asymptomatic carriers with different *SDHx* mutations aims to unify global clinical practice. It also provides guidance for clinicians that can be adapted to their own and their patient’s situations. Many outstanding issues, such as age at first screening during childhood, optimal time period between two assessments or the need for a different approach according to mutation subtypes, support the need for large, international prospective studies in the near future as the effect of an early diagnosis on outcome needs to be balanced with the burden and the costs of screening. This screening protocol based on expert opinion should be regularly reviewed and updated when more conclusive data become available.

## Supplementary information

Supplementary Information
