# Supplementary material for: International consensus on initial screening and follow-up of asymptomatic SDHx mutation carriers
Source: Nat Rev Endocrinol. 2021 May 21;17(7):435–44. doi: 10.1038/s41574-021-00492-3 (PMC8205850; doi:10.1038/s41574-021-00492-3)
Supplement: Supplementary file 1 — Supplementary Information [file 41574_2021_492_MOESM1_ESM.pdf]

nature portfolio

https://doi.org/10.1038/s41574-021-00492-3

Supplementary information

International consensus on initial screening and follow-up of asymptomatic *SDHx* mutation carriers

In the format provided by the authors and unedited

Table S1: First round DELPHI questionnaire

I. **Initial screening**

Initial screening is defined as the time point at which the mutation has been diagnosed for the first time

1. For whom would you perform a tumour screening after an *SDHx* mutation discovery in a relative subject?
    - o *SDHB* mutation carrier
    - o *SDHC* mutation carrier
    - o *SDHA* mutation carrier
    - o *SDHD* mutation carrier father inherited
    - o *SDHD* mutation carrier mother inherited
2. If you have ticked a box in the precedent question, at which age would you perform the first screening

	SDHA	*SDHB*	*SDHC*	*SDHD* father inherited	*SDHD* mother maternally
Before 6 years old					
Between 6 and 10 years old					
Between 11 and 15 years old					
After 16 years old					
After 18 years old					
Not enough evidence to answer this question					

INITIAL SCREENING DURING CHILDHOOD *(age< 18 years old)*

If you screen these subjects, how would you proceed during Childhood (age< 18 years old)?

3. Clinically including
    - o Out-of-office Blood Pressure measurement
    - o Symptoms questionnaire
4. Biochemistry
    - o No
    - o Yes
    - o No opinion
5. If yes, what type?
    - ▪ Plasma free normetanephrine
    - ▪ Plasma free metanephrine
    - ▪ Plasma methoxytyramine
    - ▪ Plasma dopamine
    - ▪ Plasma catecholamines
    - ▪ Plasma VMA

- Plasma chromogranine A
- Urinary fractionated normetanephrine
- Urinary fractionated metanephrine
- Urinary methoxytyramine
- Urinary dopamine
- Urinary catecholamines
- Urinary VMA

6. Any Anatomic imaging (if yes, please fill the table below)
   - No
   - Yes
   - No opinion
7. Any Functional imaging (if yes, please fill the table below)
   - Yes
   - No
   - No opinion
8. If yes, please fill the following table

	SDHA	*SDHB*	*SDHC*	*SDHD* father inherited	*SDHD* maternally inherited
No imaging before 18 years old					
HαN conventional imaging (by US ,CT scan or MRI)					
Thoracic conventional imaging (by US ,CT scan or MRI)					
Abdomino-pelvic conventional imaging (by US ,CT scan or MRI)					
^{123}I-MIBG scintigraphy					
111In –pentetreotide scintigraphy					
^{18}F-FDG- PET/CT					
^{18}F-DOPA PET/CT					
^{68}Ga-DOTA SSA PET/CT					
At least an association of one conventional and one functional imaging					
No opinion					

9. If your answer was “yes” for anatomic imaging, what would have been your first choice

	Head and neck	Thorax	Abdomen and pelvis
MRI with contrast agent			

MRI without contrast agent			
CT with contrast agent			
CT without contrast agent			
Ultrasound			
No opinion			

INITIAL SCREENING DURING ADULTHOOD ***(age ≥ 18 years old)***

If you screen these subjects, how would you proceed during adulthood (age ≥ 18 years old)?

10. Clinically including
    - Out-of-office Blood Pressure
    - Symptoms questionnaire
11. Biochemistry
    - No
    - Yes
    - No opinion
12. If yes, what type?
    - Plasma free normetanephrine
    - Plasma free metanephrine
    - Plasma methoxytyramine
    - Plasma dopamine
    - Plasma catecholamines
    - Plasma VMA
    - Plasma chromogranine A
    - Urinary fractionated normetanephrine
    - Urinary fractionated metanephrine
    - Urinary methoxytyramine
    - Urinary dopamine
    - Urinary catecholamines
    - Urinary VMA
13. Anatomic imaging
    - No
    - Yes
    - No opinion
14. Functional imaging
    - Yes
    - No
    - No opinion
15. If yes, please fill the following table

	SDHA	*SDHB*	*SDHC*	*SDHD* father inherited	*SDHD* maternally inherited
No imaging for initial screening					

HαN conventional imaging					
Thoracic conventional imaging					
Abdomino-pelvic conventional imaging					
^{123}I-MIBG scintigraphy					
^{111}In –pentetreotide scintigraphy					
^{18}F-FDG-PET/CT					
^{18}F-DOPA PET/CT					
^{68}Ga-DOTA -SSA PET/CT					
At least an association of one conventional and one functional imaging					
No opinion					

16. If your answer was "yes" for anatomic imaging, what would have been your first choice

	Head and neck	Thorax	Abdomen and pelvis
MRI with contrast agent			
MRI without contrast agent			
CT with contrast agent			
CT without contrast agent			
Ultrasound			
No opinion			

II. Follow-up after a negative initial work-up

17. Who would you follow?
    - o *SDHB* asymptomatic mutation carrier
    - o *SDHC* asymptomatic mutation carrier
    - o *SDHA* asymptomatic mutation carrier
    - o *SDHD* asymptomatic mutation carrier father inherited,
    - o *SDHD* asymptomatic mutation carrier maternally inherited
    - o Nobody

<u>FOLLOW-UP DURING CHILDHOOD (*age< 18 years old*)</u>

18. Would you perform a regular clinical follow-up (defined as symptoms questionnaire and BP measurement)?
    - o No
    - o Yes
19. If yes: when? please fill the following table

	SDHA	*SDHB*	*SDHC*	*SDHD* father inherited	*SDHD* mother inherited

No need for follow-up					
Every year					
Every 2 years					
Every 3 years					
Every 5 years					
Every 10 years					
No opinion					

20. Do you think that a regularly Biochemistry follow-up should be performed?
    - No
    - Yes
    - No opinion
21. If yes: when? please fill the following table

	SDHA	*SDHB*	*SDHC*	*SDHD* father inherited	*SDHD* mother inherited
No need for follow-up					
Every year					
Every 2 years					
Every 3 years					
Every 5 years					
Every 10 years					
No opinion					

22. Do you think that the biochemistry assessment should be different from the initial screening?
    - No
    - Yes
    - No opinion

23. Do you think that we should perform an imaging follow-up on a regular basis?
    - No
    - Yes
    - No opinion
24. If yes, when? please fill the following table

	SDHA	*SDHB*	*SDHC*	*SDHD* father inherited	*SDHD* maternally inherited
No need for follow-up					
Every year					
Every 2 years					

Every 3 years					
Every 5 years					
Every 10 years					
No opinion					

25. If yes, should we perform
    - Conventional imaging only?
    - Functional imaging only?
    - An association of both conventional and functional imaging at each work-up?
    - Would you alternate conventional and functional imaging?

26. If yes, please fill the following table

	SDHA	*SDHB*	*SDHC*	*SDHD* father inherited	*SDHD* maternally inherited
HαN conventional imaging					
Thoracic conventional imaging					
Abdomino-pelvic conventional imaging					
^{123}I-MIBG scintigraphy					
^{111}In –pentetreotide scintigraphy					
^{18}F-FDG-PET/CT					
^{18}F-DOPA PET/CT					
^{68}Ga-DOTA -SSA PET/CT					
At least an association of one conventional and one functional imaging					
No opinion					

27. If your answer was "yes" for anatomical imaging, what would have been your first choice

	Head and neck	Thorax	Abdomen and pelvis
MRI with contrast agent			
MRI without contrast agent			
CT with contrast agent			
CT without contrast agent			
Ultrasound			
No opinion			

FOLLOW-UP DURING ADULTHOOD (*age ≥ 18 years old*)

28. Would you perform a regular clinical follow-up (defined as symptoms questionnaire and BP measurement)?
    - No
    - Yes
29. If yes: when? please fill the following table

	SDHA	*SDHB*	*SDHC*	*SDHD* father inherited	*SDHD* maternally inherited
No need for follow-up					
Every year					
Every 2 years					
Every 3 years					
Every 5 years					
Every 10 years					

30. Do you think that a regularly Biochemistry follow-up should be performed?
    - No
    - Yes
    - No opinion
31. If yes: when? please fill the following table

	SDHA	*SDHB*	*SDHC*	*SDHD* father inherited	*SDHD* mother inherited
No need for follow-up					
Every year					
Every 2 years					
Every 3 years					
Every 5 years					
Every 10 years					

32. Do you think that the biochemistry assessment should be different from the initial screening?
    - No
    - If yes, please comment
    - No opinion

33. Do you think that we should perform an imaging follow-up on a regular basis?
    - No

- Yes
- No opinion

34. If yes, when? please fill the following table

	SDHA	*SDHB*	*SDHC*	*SDHD* father inherited	*SDHD* maternally inherited
No need for follow-up					
Every year					
Every 2 years					
Every 3 years					
Every 5 years					
Every 10 years					

35. If yes, should we perform
    - Conventional imaging only?
    - Functional imaging only?
    - An association of both conventional and functional imaging at each work-up?
    - Would you alternate conventional and functional imaging?

36. If yes, please fill the following table

	SDHA	*SDHB*	*SDHC*	*SDHD* father inherited	*SDHD* maternally inherited
No imaging for Follow-up					
HαN conventional imaging					
Thoracic conventional imaging					
Abdomino-pelvic conventional imaging					
^{123}I-MIBG scintigraphy					
^{111}In –pentetreotide scintigraphy					
^{18}F-FDG-PET					
^{18}F-DOPA PET					
^{68}Ga-DOTA -somatostatin receptor PET					
At least an association of one conventional and one functional imaging					
No opinion					

37. If your answer was “yes” for anatomical imaging, what would have been your first choice

	Head and neck	Thorax	Abdomen and pelvis
MRI with contrast agent			
MRI without contrast agent			
CT with contrast agent			
CT without contrast agent			
Ultrasound			
No opinion			

END OF FOLLOW UP

38. Do you think that we should stop the follow-up from a certain age?
    - No
    - Yes, After 60
    - Yes, After 70
    - Yes, After 80
    - Yes, After 90
    - No opinion

39. Do you think that we should reduce the follow-up from a certain age?
    - No
    - Yes, After 60
    - Yes, After 70
    - Yes, After 80
    - Yes, After 90
    - No opinion
40. If yes, should the frequency be reduced?
    - No
    - Yes
    - No opinion
41. If yes, what would be the ideal frequency then (in years)?
42. If yes how should the follow-up be reduced? please fill the following table

	SDHA	*SDHB*	*SDHC*	*SDHD* father inherited	*SDHD* maternally inherited
Clinical follow-up					
Biochemistry					
Conventional imaging					
Functional imaging					

III. Additionally questions

43. Do you consider that initial screening and/or follow-up should be different for asymptomatic carriers whose family members developed metastatic SDHx-related PPGL vs those with non-metastatic?
44. Do you consider that initial screening and/or follow-up should be different to females vs males?
45. Do you consider that environmental factors should be taken into account: e.g. somebody living in a high altitude?
46. Do you consider that screening for other tumours commonly found in *SDHx* carriers should be done?
    - No
    - Yes, for RCC
    - Yes, for GIST
    - Yes, for pituitary adenoma
    - Yes, for another tumour
    - No opinion
47. If yes, does this apply:
    - only to the first screening
    - on a regular basis, at the same time interval as for PPGL
    - on a regular basis, at a different time interval then for PPGL
    - No opinion

IV. Would you like to discuss some other point in this consensus?

If yes, please comment

Table S2: Second round DELPHI questionnaire

I. Initial screening

Initial screening is defined as the time point at which the mutation was diagnosed for the first time

1. Tumour screening should be performed after an *SDHA, SDHB, SDHC* and *SDHD-fi* mutation discovery in a relative
   - Strongly agree
   - Agree
   - Neutral
   - Disagree
   - Strongly disagree

2. Are you in agreement that in childhood tumour screening should only be performed following positive discovery of a mutation?
   - Strongly agree
   - Agree
   - Neutral
   - Disagree
   - Strongly disagree

3. No tumour screening should be performed before the age of 6 in an asymptomatic *SDHx* mutation carrier.
   - Strongly agree
   - Agree
   - Neutral
   - Disagree
   - Strongly disagree

4. Tumour screening should be performed before the age of 16 after in an asymptomatic *SDHB* mutation carrier.
   - Strongly agree
   - Agree
   - Neutral
   - Disagree
   - Strongly disagree

5. Do you think that the first screening in an asymptomatic *SDHB* mutation carrier should be performed:
   - between 6 and 10 yo
   - between 11 and 15 yo

- between 6 and 15 yo
- No opinion

6. Could you please complete the Table:

	SDHA	*SDHC*	*SDHD fi*
Between 6 and 10 years old			
Between 11 and 15 years old			
Between 6 and 15 years old			
After 16 years old			
After 18 years old			
No opinion			

<u>IF YOU SCREEN THESE SUBJECTS, HOW WOULD YOU PROCEED DURING CHILDHOOD (*age < 18 years old*)?</u>

7. Tumour screening in *SDHA, SDHB, SDHC and SDHD fi* asymptomatic mutation carriers should include clinical examination (blood Pressure measurement, ideally out-of office, and a symptoms questionnaire)
    - Strongly agree
    - Agree
    - Neutral
    - Disagree
    - Strongly disagree

8. Tumour screening in *SDHA, SDHB, SDHC and SDHD fi* asymptomatic mutation carriers should include biochemistry
    - Strongly agree
    - Agree
    - Neutral
    - Disagree
    - Strongly disagree

9. During Childhood, if a tumour screen is performed, biochemistry testing should not rely on the assessment of plasma or urinary dopamine, catecholamines, VMA, or plasma chromogranin A.
    - Strongly agree
    - Agree
    - Neutral
    - Disagree
    - Strongly disagree

10. During childhood, plasma free metanephrine and free normetanephrine should be performed for initial screening.
    - Strongly agree
    - Agree
    - Neutral
    - Disagree
    - Strongly disagree

11. Do you consider that urinary metanephrine and urinary normetanephrine should be performed:
    - In addition to the assessment of plasma metanephrine/normetanephrine
    - Only if plasma free metanephrine/normetanephrine are not available
    - They shouldn't be performed.
    - No opinion

12. Do you consider that plasma methoxytyramine should be performed:
    - In addition to the assessment of plasma metanephrine/normetanephrine
    - They shouldn't be performed in asymptomatic subjects.
    - No opinion

13. Do you consider that urinary methoxytyramine should be performed:
    - In addition to the assessment of plasma metanephrine/normetanephrine
    - Only in addition to the assessment of urinary metanephrine/normetanephrine if they are performed
    - They shouldn't be performed in asymptomatic subjects.
    - No opinion

14. Tumour screening in *SDHA, SDHB, SDHC and SDHD fi* asymptomatic mutation carriers during childhood should include anatomical imaging
    - Strongly agree
    - Agree
    - Neutral
    - Disagree
    - Strongly disagree

15. Ultrasound should not be used for initial tumour screening in *SDHA, SDHB, SDHC and SDHD fi* asymptomatic mutation carrier during childhood.
    - Strongly agree
    - Agree
    - Neutral
    - Disagree
    - Strongly disagree

16. Please complete the following table for *SDHA*.

SDHA mutation	*MRI*	*CT*	*Nothing*	*No opinion*
Head and Neck				
Thoracic				
Abdomino-pelvic				

17. Please complete the following table for *SDHB*

SDHB mutation	*MRI*	*CT*	*Nothing*	*No opinion*
Head and Neck				
Thoracic				
Abdomino-pelvic				

18. Please complete the following table for *SDHC*

SDHC mutation	*MRI*	*CT*	*Nothing*	*No opinion*
Head and Neck				
Thoracic				
Abdomino-pelvic				

19. Please complete the following table for *SDHD fi*

SDHD FI mutation	*MRI*	*CT*	*Nothing*	*No opinion*
Head and Neck				
Thoracic				
Abdomino-pelvic				

20. Tumour screening in *SDHA, SDHB, SDHC and SDHD fi* asymptomatic mutation carriers during **childhood** should include functional imaging for **initial screening**
    - Strongly agree
    - Agree
    - Neutral
    - Disagree
    - Strongly disagree

IF YOU SCREEN THESE SUBJECTS, HOW WOULD YOU PROCEED DURING ADULTHOOD (*age ≥ 18 years old*)?

21. Initial tumour screening in *SDHA, SDHB, SDHC* and *SDHD fi* asymptomatic mutation carrier during adulthood should include clinical examination (blood pressure measurement, ideally out-of office, and a symptoms questionnaire), biochemistry and anatomical imaging.
    - Strongly agree
    - Agree
    - Neutral
    - Disagree
    - Strongly disagree

22. During adulthood, if a tumour screen is performed, biochemistry testing should include plasma free metanephrine and free normetanephrine.
    - Strongly agree
    - Agree
    - Neutral
    - Disagree
    - Strongly disagree

23. During adulthood, if a tumour screen is performed, the biochemistry testing should not rely on the assessment of plasma or urinary dopamine, catecholamines, VMA, plasma chromogranin A, or urinary methoxytyramine.
    - Strongly agree
    - Agree
    - Neutral
    - Disagree
    - Strongly disagree

24. Do you think that urinary metanephrine and urinary normetanephrine should be performed:
    - In addition to the assessment of plasma metanephrine/normetanephrine
    - Only if plasma free metanephrine/normetanephrine are not available
    - They shouldn't be performed.
    - No opinion

25. Do you think that plasma methoxytyramine should be performed:
    - In addition to the assessment of plasma metanephrine/normetanephrine
    - It shouldn't be performed for asymptomatic subjects.
    - No opinion

26. Do you think that urinary methoxytyramine should be performed:
    - In addition to the assessment of plasma metanephrine/normetanephrine
    - Only in addition to the assessment of urinary metanephrine/normetanephrine
    - It shouldn't be performed in asymptomatic subjects.

- No opinion

27. Ultrasound should not be used for initial tumour screening in *SDHA*, *SDHB*, *SDHC and SDHD fi* asymptomatic mutation carriers during adulthood
    - Strongly agree
    - Agree
    - Neutral
    - Disagree
    - Strongly disagree

28. ^{123}I-MIBG should not be used for initial tumour screening in *SDHA*, *SDHB*, *SDHC and SDHD fi* asymptomatic mutation carriers during adulthood
    - Strongly agree
    - Agree
    - Neutral
    - Disagree
    - Strongly disagree

29. Tumour screening in *SDHA*, *SDHB*, *SDHC* and *SDHD fi* asymptomatic mutation carriers during adulthood should include functional imaging for initial screening
    - Strongly agree
    - Agree
    - Neutral
    - Disagree
    - Strongly disagree

30. Please complete the table below for *SDHA*

SDHA	*Strongly Agree*	*Agree*	*Neutral*	*Disagree*	*Strongly Disagree*
^{18}F-FDG-PET/CT					
^{18}F-DOPA PET/CT					
^{68}Ga-DOTA-SSA PET/CT					
At least one Functional imaging					

31. Please complete the table below for *SDHB*

SDHB	*Strongly Agree*	*Agree*	*Neutral*	*Disagree*	*Strongly Disagree*
^{18}F-FDG-PET/CT					

^{18}F-DOPA PET/CT					
^{68}Ga-DOTA-SSA PET/CT					
At least one Functional imaging					

32. Please complete the table below for *SDHC*

SDHC	*Strongly Agree*	*Agree*	*Neutral*	*Disagree*	*Strongly Disagree*
^{18}F-FDG-PET/CT					
^{18}F-DOPA-PET/CT					
^{68}Ga-DOTA-SSA PET/CT					
At least one Functional imaging					

33. Please complete the table below for *SDHD FI*

SDHD fi	*Strongly Agree*	*Agree*	*Neutral*	*Disagree*	*Strongly Disagree*
^{18}F-FDG-PET/CT					
^{18}F-DOPA-PET/CT					
^{68}Ga-DOTA-SSA PET/CT					
At least one Functional imaging					

34. Please complete the following table for *SDHA*

SDHA mutation	*MRI*	*CT*	*Nothing*	*No opinion*
HαN				
Thoracic				
Abdomino-pelvic				

35. Please complete the following table for *SDHB*

SDHB mutation	*MRI*	*CT*	*Nothing*	*No opinion*
HαN				
Thoracic				
Abdomino-pelvic				

36. Please complete the following table for *SDHC*

SDHC mutation	*MRI*	*CT*	*Nothing*	*No opinion*
HαN				
Thoracic				
Abdomino-pelvic				

37. Please complete the following table for *SDHD fi*

SDHD fi mutation	*MRI*	*CT*	*Nothing*	*No opinion*
HαN				
Thoracic				
Abdomino-pelvic				

II. Follow-up after a NEGATIVE Initial work-up

WHO WOULD YOU FOLLOW?

38. *SDHB*, *SDHC and SDHD*-fi asymptomatic mutation carriers should be followed on a regular basis even after a negative initial work-up
    - Strongly agree
    - Agree
    - Neutral
    - Disagree
    - Strongly disagree

39. A regular follow-up should be performed after a negative initial work-up for *SDHA* asymptomatic mutation carriers?
    - Strongly agree
    - Agree
    - Neutral
    - Disagree
    - Strongly disagree

HOW WOULD YOU FOLLOW THE ASYMPTOMATIC CARRIERS DURING CHILDHOOD? (age < 18 years old)

40. Follow-up of *SDHA*, *SDHB*, *SDHC* and *SDHD fi* mutation carriers during childhood should include clinical examination (blood pressure measurement, ideally out-of office, and a symptoms questionnaire), the same biochemical investigations as the initial screen and imaging
    - Strongly agree
    - Agree
    - Neutral
    - Disagree
    - Strongly disagree

41. Please complete the following table for *SDHB*

SDHB mutation	*Never*	*Every year*	*Every 2 years*	*Every 3 years*	*Every 5 years*	*No Opinion*
Clinical						
Biological						
Imaging						

42. Please complete the following table for *SDHC*

SDHC mutation	*Never*	*Every year*	*Every 2 years*	*Every 3 years*	*Every 5 years*	*No Opinion*
Clinical						
Biological						
Imaging						

43. Please complete the following table for *SDHD fi*

SDHD fi mutation	*Never*	*Every year*	*Every 2 years*	*Every 3 years*	*Every 5 years*	*No Opinion*
Clinical						
Biological						
Imaging						

44. At least one functional imaging should be performed during follow-up in childhood:
    - Strongly agree
    - Agree
    - Neutral
    - Disagree
    - Strongly disagree

45. Ultrasound should not be used for follow-up during childhood in *SDHB, SDHC* and *SDHD fi* asymptomatic mutation carriers during childhood
    - Strongly agree
    - Agree
    - Neutral
    - Disagree
    - Strongly disagree

46. Please complete the table below for *SDHB*

SDHB mutation	*MRI*	*CT*	*Nothing*	*No opinion*
Head and Neck				
Thoracic				
Abdomino-pelvic				

47. Please complete the table below for *SDHC*

SDHB mutation	*MRI*	*CT*	*Nothing*	*No opinion*
Head and Neck				
Thoracic				
Abdomino-pelvic				

48. Please complete the table below for *SDHD fi*

SDHC mutation	*MRI*	*CT*	*Nothing*	*No opinion*
Head and Neck				
Thoracic				
Abdomino-pelvic				

HOW WOULD YOU FOLLOW ASYMPTOMATIC MUTATION CARRIERS DURING ADULTHOOD? (*age ≥ 18 years old*)

49. Follow-up of *SDHB, SDHC* and *SDHD-fi* asymptomatic mutation carriers during adulthood should include clinical examination (blood pressure measurement, ideally out-of office, and a symptoms questionnaire), the same biochemical investigations as at the initial screening and imaging
    - Strongly agree
    - Agree
    - Neutral

- Disagree
- Strongly disagree

50. Please complete the following table for SDHB

SDHB mutation	*Never*	*Every year*	*Every 2 years*	*Every 3 years*	*No Opinion*
Clinical					
Biological					
Imaging					

51. Please complete the following table for SDHC

SDHC mutation	*Never*	*Every year*	*Every 2 years*	*Every 3 years*	*Every 5 years*	*No Opinion*
Clinical						
Biological						
Imaging						

52. Please complete the following table for *SDHD fi*

SDHD fi mutation	*Never*	*Every year*	*Every 2 years*	*Every 3 years*	*Every 5 years*	*No Opinion*
Clinical						
Biological						
Imaging						

53. At least one functional imaging should be performed during the follow-up in adulthood:
    - Strongly agree
    - Agree
    - Neutral
    - Disagree
    - Strongly disagree

54. Ultrasound should not be used for initial tumour screening in *SDHB, SDHC and SDHD fi* asymptomatic mutation carriers during adulthood
    - o Strongly agree
    - o Agree
    - o Neutral
    - o Disagree
    - o Strongly disagree

55. Please complete the table below for *SDHB*

SDHB mutation	*MRI*	*CT*	*Nothing*	*No opinion*
Head and Neck				
Thoracic				
Abdomino-pelvic				

56. Please complete the table below for *SDHC*

SDHC mutation	*MRI*	*CT*	*Nothing*	*No opinion*
Head and Neck				
Thoracic				
Abdomino-pelvic				

57. Please complete the table below for *SDHD FI*

SDHC mutation	*MRI*	*CT*	*Nothing*	*No opinion*
Head and Neck				
Thoracic				
Abdomino-pelvic				

58. A different type of imaging (ie CT or functional imaging) could be chosen depending on the age of the patient:
    - o Strongly agree
    - o Agree
    - o Neutral
    - o Disagree
    - o Strongly disagree

If yes, can you please say at what age?

END OF FOLLOW-UP

59. Follow-up should be stopped after 80 years old
    - Strongly agree
    - Agree
    - Neutral
    - Disagree
    - Strongly disagree

60. Follow-up should be reduced after 60 years old
    - Strongly agree
    - Agree
    - Neutral
    - Disagree
    - Strongly disagree

61. Follow-up should be reduced to every 5 years from 60 years old
    - Strongly agree
    - Agree
    - Neutral
    - Disagree
    - Strongly disagree

62. No consensus was reached regarding changes of screening methods from a certain age. Therefore we suggest not raising this point in the consensus. Do you agree?
    - Strongly agree
    - Agree
    - Neutral
    - Disagree
    - Strongly disagree

III. *SDHD* Maternally Inherited

63. A tumour screening should be performed in an *SDHD-maternally inherited (mi)* asymptomatic mutation carrier?
    - Strongly agree
    - Agree
    - Neutral
    - Disagree

- Strongly disagree

64. Do you think that first screening in an *SDHD-mi* asymptomatic mutation carrier should be performed:
    - between 6 and 10 yo
    - between 11 and 15 yo
    - between 6 and 15 yo
    - After 16 years old
    - After 18 years old
    - No opinion
    - No screening

65. A regular follow-up should be performed after a negative initial work-up for *SDHD-mi* asymptomatic mutation carriers.
    - Strongly agree
    - Agree
    - Neutral
    - Disagree
    - Strongly disagree

IV. Additionally questions

66. Initial screening and/or follow-up should be different for asymptomatic carriers whose family members developed metastatic *SDHx*-related PPGL versus those with non-metastatic
    - Strongly agree
    - Agree
    - Neutral
    - Disagree
    - Strongly disagree

67. So, do you consider that a complete screening should be performed before planning a pregnancy?
    - Strongly agree
    - Agree
    - Neutral
    - Disagree
    - Strongly disagree

68. Environmental factors should be taken into account: e.g. somebody living in a high altitude
    - Strongly agree
    - Agree
    - Neutral
    - Disagree
    - Strongly disagree

69. Do you consider that additional imaging should be performed to screen for other tumours. Please the table below:

	RCC	GIST	Pituitary adenoma
Yes			
No			
No, same imaging as for PPGL			
No but the radiologist should be informed			
No opinion			

Table S3: third round DELPHI questionnaire

I. Initial screening

1. During childhood, do you agree that genetic screening should only be performed if a tumour screening is considered in case of positive status?
    - Strongly agree
    - Agree
    - Neutral
    - Disagree
    - Strongly disagree

2. Do you think that first tumour screening in an asymptomatic *SDHB* mutation carrier should be performed between 6 and 10 years old?
    - Strongly agree
    - Agree
    - Neutral
    - Disagree
    - Strongly disagree

3. Do you agree that first tumour screening should be performed before the age of 16 in an asymptomatic *SDHA*, *SDHC* or *SDHD-fi* mutation carrier?
    - Strongly agree
    - Agree
    - Neutral
    - Disagree
    - Strongly disagree

4. Do you agree that first tumour screening should be performed between 11 and 15 years old in an asymptomatic *SDHA* mutation carrier?
    - Strongly agree
    - Agree
    - Neutral
    - Disagree
    - Strongly disagre

5. Do you think that the first screening in an asymptomatic *SDHC* mutation carrier should be performed:
    - between 6 and 10 yo
    - between 11 and 15 yo
    - between 6 and 15 yo

- No opinion

6. Do you think that the first screening in an asymptomatic *SDHD-fi* mutation carrier should be performed
   - between 6 and 10 yo
   - between 11 and 15 yo
   - between 6 and 15 yo
   - No opinion

INITIAL SCREENING DURING ADULTHOOD *(age < 18 years old)*

7. Do you consider that urinary metanephrine and urinary normetanephrine should be performed only if plasma metanephrine/normetanephrine are not available?
   - Strongly agree
   - Agree
   - Neutral
   - Disagree
   - Strongly disagree

8. During Childhood, if a tumour screening is performed, biochemistry testing should include the following hormones in addition to metanephrine and normetanephrine: please fill the table below

Hormone	*Strongly Agree*	*Agree*	*Neutral*	*Disagree*	*Strongly disagree*
Urinary dopamine					
Plasma dopamine					
Catecholamines					
Vanillylmandelic acid (VMA)					
Plasma chromogranin A					

9. During childhood, do you consider that plasma methoxytyramine should be performed in addition to the assessment of plasma metanephrine and normetanephrine?
   - Strongly agree
   - Agree
   - Neutral
   - Disagree
   - Strongly disagree

10. During childhood, do you consider that urinary methoxytyramine should NOT be performed in addition to the assessment of metanephrine and normetanephrine? (please pay attention to the negative formulation)
    - Strongly agree
    - Agree
    - Neutral
    - Disagree
    - Strongly disagree

11. During childhood, MRI should be used as first line imaging for initial thoracic tumour screening in asymptomatic *SDHA*, *SDHB*, *SDHC* and *SDHD-fi* mutation carriers. Do you agree?
    - Strongly agree
    - Agree
    - Neutral
    - Disagree
    - Strongly disagree

12. During childhood, MRI should be used as first line imaging for initial abdomino-pelvic tumour screening in *SDHA*, *SDHB*, *SDHC* and *SDHD-fi* asymptomatic mutation carriers. Do you agree?
    - Strongly agree
    - Agree
    - Neutral
    - Disagree
    - Strongly disagree

13. During childhood, ultrasound should NOT be used as first line imaging for initial tumour screening in asymptomatic *SDHA*, *SDHB*, *SDHC* and *SDHD-fi* mutation carriers (pay attention to the negative formulation). Do you agree?
    - Strongly agree
    - Agree
    - Neutral
    - Disagree
    - Strongly disagree

14. Do you consider that functional imaging should NOT be used for tumour screening as first line imaging in asymptomatic SDH mutation carriers during childhood? (pay attention to the negative formulation).
    - Strongly agree
    - Agree
    - Neutral
    - Disagree
    - Strongly disagree

INITIAL SCREENING DURING ADULTHOOD *(age ≥ 18 years old)*

15. During Adulthood, do you consider that urinary metanephrine and urinary normetanephrine should be performed only if plasma free metanephrine and normetanephrine are not available?
    - Strongly agree
    - Agree
    - Neutral
    - Disagree
    - Strongly disagree

16. During Adulthood, if a tumour screening is performed, biochemistry testing should include the following hormones in addition to metanephrine and normetanephrine: please fill the table below

Hormone	*Strongly Agree*	*Agree*	*Neutral*	*Disagree*	*Strongly disagree*
Urinary dopamine					
Plasma dopamine					
Catecholamines					
Vanillylmandelic acid (VMA)					
Plasma chromogranin A					

17. During adulthood, do you consider that plasma methoxytyramine should be performed in addition to the assessment of plasma free metanephrine and normetanephrine?
    - Strongly agree
    - Agree
    - Neutral
    - Disagree
    - Strongly disagree

18. During adulthood, do you consider that urinary methoxytyramine should NOT be performed in asymptomatic SDH mutation carriers (please pay attention to the negation in the formulation)
    - Strongly agree
    - Agree
    - Neutral
    - Disagree
    - Strongly disagree

19. During adulthood, MRI should be used as first line imaging for initial thoracic tumour screening in asymptomatic *SDHA*, *SDHB*, *SDHC* and *SDHD-fi* mutation carriers. Do you agree?
    - Strongly agree
    - Agree
    - Neutral
    - Disagree
    - Strongly disagree

20. During adulthood, MRI should be used as first line imaging for initial abdomino-pelvic tumour screening in asymptomatic *SDHA*, *SDHB*, *SDHC* and *SDHD-fi* mutation carriers. Do you agree?
    - Strongly agree
    - Agree
    - Neutral
    - Disagree
    - Strongly disagree

21. Tumour screening should include functional imaging at least once for initial screening in asymptomatic *SDHA*, *SDHB*, *SDHC* and *SDHD-fi* mutation carriers during adulthood.
    - Strongly agree
    - Agree
    - Neutral
    - Disagree
    - Strongly disagree

22. Considering disparity in availability, cost and habitus, we suggest not raising the question of the type of PET tracer in the consensus. Do you agree?
    - Strongly agree
    - Agree
    - Neutral
    - Disagree
    - Strongly disagree

23. Regarding the answers and comments to previous questions on imaging screening. Do you agree that first initial screening in asymptomatic *SDHA*, *SDHB*, *SDHC* and *SDHD-fi* mutation carriers would ideally benefit from an association of head and neck MRI, thoraco-abdminopelvic MRI (or whole body MRI) and a PET CT, during adulthood?
    - Strongly agree
    - Agree
    - Neutral
    - Disagree

- Strongly disagree

III. Follow-up after a negative Initial work-up

FOLLOW-UP DURING CHILDHOOD (age < 18 years old)

24. During childhood, follow-up of *SDHC* and *SDHD-fi* asymptomatic mutation carrier should include clinical examination (blood pressure measurement, ideally out-of office, and a symptoms questionnaire) every year. Do you agree?
    - Strongly agree
    - Agree
    - Neutral
    - Disagree
    - Strongly disagree

25. During childhood, follow-up of asymptomatic *SDHB*, *SDHC* and *SDHD-fi* mutation carriers should include biochemistry (ie metanephrine and normetanephrine) at least every two years. Do you agree?
    - Strongly agree
    - Agree
    - Neutral
    - Disagree
    - Strongly disagree

26. During childhood, follow-up of asymptomatic *SDHB*, *SDHC* and *SDHD*-fi mutation carriers should include imaging every 2 to 3 years. Do you agree?
    - Strongly agree
    - Agree
    - Neutral
    - Disagree
    - Strongly disagree

27. Do you consider that functional imaging should NOT be used for follow-up as first line imaging in asymptomatic *SDHB*, *SDHC*, *SDHD-fi* mutation carriers during childhood? (pay attention to the negative formulation).
    - Strongly agree
    - Agree
    - Neutral
    - Disagree
    - Strongly disagree

28. During childhood, MRI should be used as first line thoracic imaging for follow-up in asymptomatic *SDHB*, *SDHC* and *SDHD-fi* mutation carriers. Do you agree?
    - Strongly agree
    - Agree
    - Neutral
    - Disagree
    - Strongly disagree

29. During childhood, MRI should be used as first line abdomino-pelvic imaging for follow-up in asymptomatic *SDHB*, *SDHC* and *SDHD-fi* mutation carriers. Do you agree?
    - Strongly agree
    - Agree
    - Neutral
    - Disagree
    - Strongly disagree

30. During childhood, asymptomatic *SDHA* mutation carriers should have the same follow-up as for the other genes. Do you agree?
    - Strongly agree
    - Agree
    - Neutral
    - Disagree
    - Strongly disagree

FOLLOW-UP DURING ADULTHOOD (*age ≥ 18 years old*)

31. During adulthood, follow-up of *SDHC* and *SDHD*-fi asymptomatic mutation carriers should include clinical examination (blood pressure measurement, ideally out-of office, and a symptoms questionnaire) every year. Do you agree?
    - Strongly agree
    - Agree
    - Neutral
    - Disagree
    - Strongly disagree

32. During adulthood, follow-up of SDHC and SDHD FI asymptomatic mutation carriers should include biochemistry (metanephrine and normetanephrine) every year. Do you agree?
    - Strongly agree
    - Agree
    - Neutral
    - Disagree
    - Strongly disagree

33. During adulthood, follow-up of asymptomatic *SDHB*, *SDHC* and *SDHD*-fi mutation carriers should include imaging every 2 to 3 years. Do you agree?
    - Strongly agree
    - Agree
    - Neutral
    - Disagree
    - Strongly disagree

34. During adulthood, MRI should be used as first line thoracic imaging for follow-up in asymptomatic *SDHB*, *SDHC* and *SDHD-fi* mutation carriers. Do you agree?
    - Strongly agree
    - Agree
    - Neutral
    - Disagree
    - Strongly disagree

35. During adulthood, MRI should be used as first line abdomino-pelvic imaging for follow-up in asymptomatic *SDHB*, *SDHC* and *SDHD*-fi mutation carriers. Do you agree?
    - Strongly agree
    - Agree
    - Neutral
    - Disagree
    - Strongly disagree

36. During adulthood, follow-up of asymptomatic SDHB, SDHC and SDHD FI mutation carriers should include functional imaging every 4 to 5 years in alternation with anatomical imaging. Do you agree?
    - Strongly agree
    - Agree
    - Neutral
    - Disagree
    - Strongly disagree

37. As no tendency emerged regarding a change in method of choice for imaging depending on the age, we suggest not to raise this point in the consensus
    - Strongly agree
    - Agree
    - Neutral
    - Disagree
    - Strongly disagree

38. During adulthood, asymptomatic *SDHA* mutation carriers should have the same follow-up as for the other genes. Do you agree?
    - Strongly agree
    - Agree
    - Neutral
    - Disagree
    - Strongly disagree

<u>END OF FOLLOW-UP</u>

39. Regarding these answers we ask the question again. If a *SDHA*, *SDHB*, *SDHC* or *SDHD-fi* mutation carrier never developed any tumour related to SDH deficiency and has been asymptomatic all life long, follow-up should be stopped at 80 years old. Do you agree?
    - Strongly agree
    - Agree
    - Neutral
    - Disagree
    - Strongly disagree

40. If an *SDHA*, *SDHB*, *SDHC* or *SDHD-fi* mutation carrier never developed any tumour related to SDH deficiency and has been asymptomatic all life long, follow-up frequency should be delayed to every 5 years after 70 years old. Do you agree?
    - Strongly agree
    - Agree
    - Neutral
    - Disagree
    - Strongly disagree

V. *SDHD* Maternally Inherited

41. In an asymptomatic *SDHD-mi* mutation carrier, screening could be performed only once, during adulthood (≥18 years old). This screening would be the same as initial screening performed for the other *SDH*x genes. Do you agree?
    - Strongly agree
    - Agree
    - Neutral
    - Disagree
    - Strongly disagree

VI. Additionally questions

42. Initial screening and/or follow-up should NOT be different for asymptomatic mutation carriers whose family members developed metastatic *SDHx*-related PPGL versus those with non-metastatic PPGL. Do you agree? (Pay attention to the negative formulation)
    - Strongly agree
    - Agree
    - Neutral
    - Disagree
    - Strongly disagree

43. As no tendency emerged regarding environmental factors, we suggest not raising this point in the consensus. Do you agree?
    - Strongly agree
    - Agree
    - Neutral
    - Disagree
    - Strongly disagree

44. No additional imaging should be performed for RCC and GIST. Nevertheless, they should be searched on imaging performed for PPGL screening. Do you agree?
    - Strongly agree
    - Agree
    - Neutral
    - Disagree
    - Strongly disagree

45. No additional imaging should be performed for pituitary adenoma screening. Do you agree?
    - Strongly agree
    - Agree
    - Neutral
    - Disagree
    - Strongly disagree

Table S4: fourth round DELPHI questionnaire

I. **Initial screening**

AGE OF FIRST SCREENING

1. First tumour screening should be performed between ages 10 and 15 years in an asymptomatic *SDHA* mutation carrier?
    - Strongly agree
    - Agree
    - Neutral
    - Disagree
    - Strongly disagree

2. First tumour screening should be performed between ages 10 and 15 years in an asymptomatic *SDHC* mutation carrier.
    - Strongly agree
    - Agree
    - Neutral
    - Disagree
    - Strongly disagree

3. The first tumour screening should be performed between ages 10 and 15 years in an asymptomatic *SDHD*-fi mutation carrier.
    - Strongly agree
    - Agree
    - Neutral
    - Disagree
    - Strongly disagree

INITIAL SCREENING DURING CHILDHOOD (*age <18 years old*)

4. Since the majority of expert opinion was neutral for dopamine, we propose not to reach this point in this consensus. To what extent do you agree with removing dopamine from further consideration?
    - Strongly agree
    - Agree
    - Neutral
    - Disagree
    - Strongly disagree

5. For methoxytyramine and Chromogranin A, we propose the statement: "The experts could not reach consensus regarding the assessment of methoxytyramine and Chromogranin A in addition to metanephrine and normetanephrine for childhood tumour screening." To what extent do you agree with this statement?
    - Strongly agree
    - Agree
    - Neutral
    - Disagree
    - Strongly disagree

<u>INITIAL SCREENING DURING ADULTHOOD (*age ≥ 18 years old*)</u>

6. During adulthood, biochemistry testing should not include Vanillylmandelic acid (VMA) nor catecholamines. (Pay attention to the negative formulation)
    - Strongly agree
    - Agree
    - Neutral
    - Disagree
    - Strongly disagree

7. For methoxytyramine and chromogranine A, we propose the statement: "Regarding the assessment of methoxytyramine and chromogranin A in addition to metanephrine and normetanephrine for tumour screening during adulthood, the experts could not reach a consensus." To what extent do you agree with this statement?
    - Strongly agree
    - Agree
    - Neutral
    - Disagree
    - Strongly disagree

8. Dopamine did not reach consensus for assessment in adulthood: to what extent do you agree with removing dopamine from further consideration?
    - Strongly agree
    - Agree
    - Neutral
    - Disagree
    - Strongly disagree

9. If PET/CT imaging does not show any thoracic abnormality, further thoracic imaging is not indicated. To what extent do you agree with this statement?
    - Strongly agree
    - Agree

- Neutral
- Disagree
- Strongly disagree
- Strongly disagree

II. Follow-up after a NEGATIVE Initial work-up

FOLLOW-UP DURING ADULTHOOD *(age ≥ 18 years old)*

10. "No consensus emerged for or against alternating PET/CT and MRI imaging during adulthood ". To what extent do you agree with this statement?
    - Strongly agree
    - Agree
    - Neutral
    - Disagree
    - Strongly disagree

III. SDHD Maternally Inherited

11. "No consensus emerged for or against the screening of asymptomatic *SDHD-mi* mutation carriers." To what extent do you agree with this statement?
    - Strongly agree
    - Agree
    - Neutral
    - Disagree
    - Strongly disagree

Abbreviations

GIST: gastro intestinal stromal tumour

PPGL: pheochromocytoma and paraganglioma

RCC: renal cell carcinoma

fi: father inherited

mi: maternally inherited

Table S5: percentage of agreement or disagreement of statements without consensus

Statement	Likert scale				
	1-Strongly Agree (%)	2-Agree (%)	3-Neutral (%)	4-Disagree (%)	5-Strongly disagree (%)
A tumour screening should be performed in an SDHD-maternally inherited (mi) asymptomatic mutation carrier.	0	48.3	20.7	27.6	3.4
Environmental factors should be taken into account: e.g. somebody living in a high altitude	3.4	10.3	37.9	41.4	6.9
During childhood, if a tumour screening is performed, biochemistry testing should include in addition to metanephrine/normetanephrine:					
➢ Urinary dopamine	6.9	3.4	37.9	27.6	24.1
➢ Plasma dopamine	3.4	6.9	24.1	37.9	27.6
➢ Plasma chromogranin A	13.8	13.8	44.8	17.2	10.3
➢ Plasma methoxytyramine	24.1	34.5	27.6	3.4	10.3
During adulthood, if a tumour screening is performed, biochemistry testing should include in addition to metanephrine/normetanephrine:					
➢ Urinary dopamine	20.7	3.4	27.6	27.6	20.7
➢ Plasma dopamine	13.8	17.2	20.7	24.1	24.1
➢ Plasma chromogranin A	17.2	24.1	41.4	6.9	10.3
➢ Plasma methoxytyramine	31	31	27.6	3.4	6.9
During adulthood, follow-up of asymptomatic mutation carriers should include functional imaging every 4 to 5 years in alternation with anatomical imaging	13.8	37.9	13.8	27.6	6.9
The PET tracer of choice for initial tumour screening during adulthood should be ^{18}F-FDG:					
➢ For *SDHA*	0	34.5	31	20.7	13.8
➢ For *SDHB*	13.8	27.6	24.1	17.2	13.8
➢ For *SDHC*	3.4	27.6	27.6	20.7	17.2
➢ For *SDHD-pi*	3.4	31	34.5	13.8	17.2
The PET tracer of choice for initial tumour screening during adulthood should be ^{18}F-FDOPA:					
➢ For *SDHA*	0	24.1	37.9	20.7	17.2
➢ For *SDHB*	0	17.2	37.9	20.7	17.2
➢ For *SDHC*	3.4	27.6	27.6	17.2	17.2

➢ For *SDHD-pi*	10.3	20.7	37.9	13.8	17.2
The PET tracer of choice for initial tumour screening during adulthood should be ^{68}Ga-DOTA-SSAs:					
➢ For *SDHA*	6.9	31.0	27.6	20.7	13.8
➢ For *SDHB*	13.8	24.1	27.6	20.7	10.3
➢ For *SDHC*	3.4	34.5	27.6	13.8	17.2
➢ For *SDHD-pi*	10.3	27.6	34.5	17.2	10.3
